# Biomechanical changes of degenerated adjacent segment and intact lumbar spine after lumbosacral topping-off surgery: a three-dimensional finite element analysis

**DOI:** 10.1186/s12891-020-3128-5

**Published:** 2020-02-15

**Authors:** Liangliang Cao, Yumei Liu, Wei Mei, Jianguang Xu, Shi Zhan

**Affiliations:** 1 Department of Spine Surgery, Zhengzhou Orthopaedics Hospital, 58 Longhai Middle Road, Zhengzhou City, Henan Province China; 2 Fudan University Shanghai Cancer Center, 270 Dong’an Road, Xuhui District, Shanghai, China; 3 Department of Spine Surgery, Shanghai Jiao Tong University Affiliated Sixth People’s Hospital, 600 Yishan Road, Xuhui District, Shanghai, China

**Keywords:** Topping-off, Finite element, Biomechanics, Fusion

## Abstract

**Background:**

Previous studies have revealed positive effect of Topping-off technique on upper adjacent segment after fusion surgery, while for the cases with fusion surgery on L5-S1 segment, owning maximal range of motion, and preexisting degenerated upper adjacent disc, it is necessary to clarify the superiority of Topping-ff technique and the effect exerted on the lumbar spine.

**Methods:**

A young healthy male volunteer was selected for thin-slice CT scanning. Then the image information was imported into the computer to establish the whole lumbar spine model as the health model. The medium degeneration model of intervertebral disc was established by changing the material properties of L4-S1 disc on the basis of the health model, and the fusion model and Topping-off model were respectively established on the basis of the degenerated model. The variation trend of ROM of L2-L5 and the stress changes of L4-L5 intervertebral disc, nucleus pulposus and facet joints were calculated respectively.

**Results:**

The L4-L5 ROM of fusion model increased significantly but the ROM of L2-L3 and L3-L4 segments did not change significantly. Compared with the degenerated model, L4-L5 activity of the Topping-off model decreased, and ROM of the L2-L3 and L3-L4 increased to some extent in the flexion and extension positions. The stress on the disc, nucleus pulposus and facet joint of the fusion model L4-L5 increased in four positions of flexion, extension, rotation and bending compared with the degenerated model, while the fiber stress on the Topping-off model decreased significantly in all four positions.

**Conclusion:**

Topping-off technology can decrease the stress and ROM of the adjacent upper degenerated segment, and increase the ROM of other upper segments, thereby protecting the degenerated upper adjacent segments and compensating the lumbar spine mobility.

## Background

In recent years, clinicians have paid more attention to the adjacent segment degeneration(ASDeg) being secondary to lumbar fusion. It is now generally accepted that increasing the fusion length promotes the occurrence of ASDeg [1–3]. In order to avoid the occurrence of ASDeg, a variety of dynamic internal fixation systems are gradually used in clinical practice, including interspinous dynamic internal fixation system, transpedicular dynamic rod fixation, artificial disc replacement, etc [4]. Although interspinous dynamic internal fixation system, to some extent, can delay the emergence of ASDeg, fusion is often required in order to achieve fully decompression and stability for patients with severe spinal stenosis or lumbar instability [5, 6]. The topping-off technique, combining lumbar fusion with the dynamic interspinous internal fixation system (Coflex), can not only provide adequate decompression to achieve good clinical efficacy but also protecting preexisting degenerated adjacent segments [7].

Several previous studies have revealed biomechanical characteristics after fusion on L3-L5 [8–10] based on healthy disc model, while fusion on L5-S1 also frequently in clinics, and considering of about 30% of the lumbar spine’s mobility existing on L5-S1, it is necessary to protect preexisting degenerated L4-L5 segment,especially for young patients. Based on the lumbar disc degeneration model, Topping-off model can provide a more accurate manifestation to the biomechanical effects on adjacent segments and entire lumbar. In addition, the supraspinal ligament was preserved and semi-laminar decompression was simulated in Topping-off model to realize highly accordance with the actual operation, and Coflex was selected as the interspinous process device.

## Methods

### Health model(HM)

Computed tomography scans of intact lumbar spine at 1-mm intervals were obtained from a healthy 25-years-old male volunteer, who was randomly selected and signed the informed consent. The FE program, ANSYS Inc. (Canonsburg, PA, USA), was used to model the spinal segments. Ligaments including ligamenta supraspinal, ligamenta interspinalia, capsular ligament, ligamentum flavum, ligamenta longitudinale posterius, ligamenta longitudinale anterius and ligamenta intertransversaria, and intervertebral disc were reconstructed according to anatomy data. The intervertebral disc and nucleus pulposus were meshed directly based on their facial meshes. The cortex was inwardly expanded by 1 mm, and then the inner side of the cortex was identified by Findface function to mesh the cancellous bone by the tetramesh function. The interface of Zygapophyseal joint was set as surface-to-surface contact with a friction coefficient of 0.1. The disc was consisted of nucleus pulposus, fibrous ring matrix and annulus fibrosus. The nucleus pulposus accounted for about 50% of the disc area and the thickness was set to 1 cm. Then the nucleus pulposus and fibrous ring matrix were both set as hyperelastic material, and the nucleus pulposus was incompressible liquid unit, while the annulus fibrosus was composed of fibrous ring matrix and collagen fiber which was simulated by two-node link elements with resistance tension only, and embedded in the fiber ring matrix with 8 layers and angles of positive-negative 30°to the end plate. The end plate with the thickness of 1 mm covered the upper and lower surface of the vertebrae. Meanwhile, the posterior facet space was set to 0.5 mm. Finally, the model was simulated according to the parameters reported in the current literature, and the specific data is shown in Table 1 [8, 9, 11–13].

**Table 1:** Material properties of the finite element model

Anatomic structure	Modulus of elasticity(MPa)	Poisson’s ratio
Osseous cortex [10]	12,000	0.3
Cancellous bone [10]	100	0.2
End plate [11]	24	0.4
Nucleus pulposus [10]	1666.7	–
Fiber ring matrix [8]	4.2	0.45
Annulus fibrosus [10]	500	0.3
Ligamenta longitudinale anteriust [9]	20	0.3
Ligamenta longitudinale posterius [9]	70	0.3
Ligamentum flavum [9–13]	50	0.3
Ligamenta interspinalia [9–13]	28	0.3
Ligamenta supraspinale [9–13]	28	0.3
Articular capsule ligament [9–13]	20	0.3
ligamenta intertransversaria [9–13]	50	0.3

### Degenerated model(DM)

Based on the healthy group model, we constructed moderate degenerated model by changing properties of annulus fibrosus and nucleus pulposus in L4-L5and L5-S1 segment [12]. Specifically, the material of nucleus pulposus was changed into a solid unit as well as the modulus of elasticity was set to 833.4 Mpa, and the elastic modulus of the fiber ring matrix was set to 8.4 Mpa.

### Fusion model(FM)

**Fig. 1:**
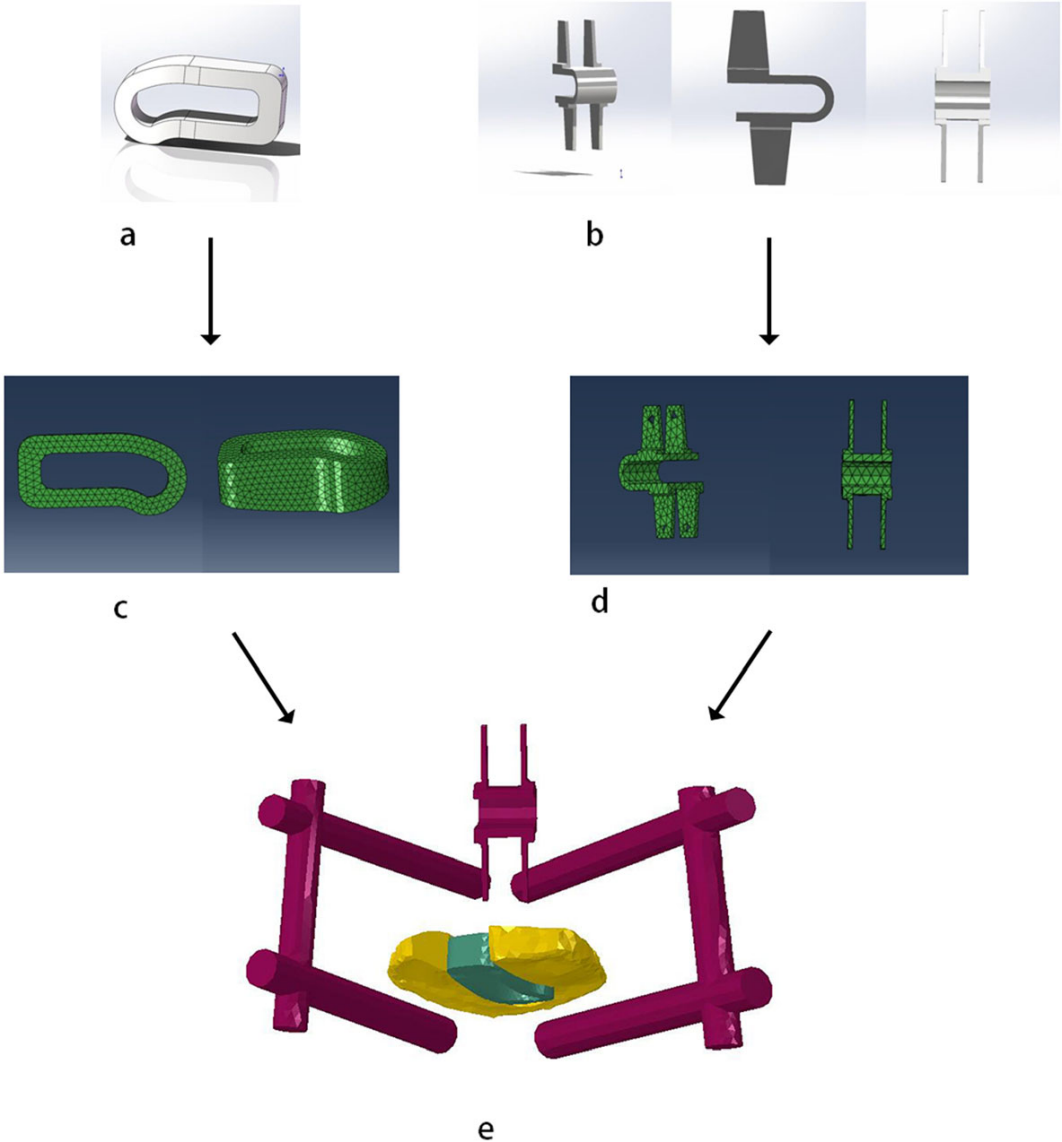
The process of establishing the internal implants models-from Constructing a geometric models of cage (**a**) and coflex (**b**) to finite element grid division (**c** and **d**), and to implants’ comnination (**e**) from Topping off model

The geometric figure of pedicle screws, rods and cage were developed in Rhinoceros 5.0 (Robert McNeil & Associates, USA) according to their parameters, and meshed with hypermesh (Fig. 1). Then these surgical instruments were assembled with the degenerated model as standard surgery, and the L5–S4 segment of the healthy model underwent partial discectomy and total nuclectomy by the posterior approach, which included removal of the semi-laminar, ipsilateral inferior articular process, posterior portions of the annulus and the entire nucleus pulposus. The elastic modulus and Poisson’s ratio of screw-rod system and cage were set as 120,000 MPa and 3600 MPa, and 1.33 and 0.38 respectively. The interfaces of screw-rod, screw-vertebra, and cage-endplate were designed to be fully constrained.

### Topping-off model(TM)

**Fig. 2:**
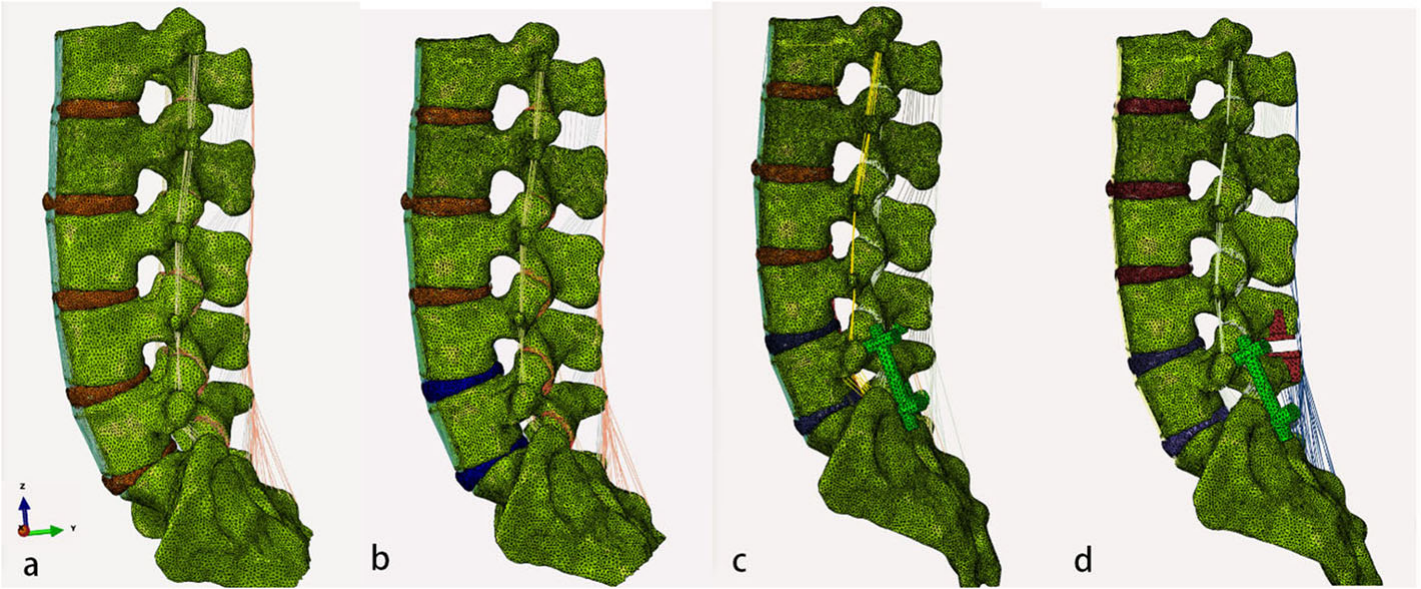
Lateral aspects of the health model (**a**), degenerated model (**b**), fusion model (**c**) and Topping-off model (**d**)

The appropriate Coflex model with the same material properties with the screw-rod system was inserted into L4–5 interspinous space of fusion model, as shown in Fig. 2. Different from the fusion model, the interspinous ligament of L4-L5 level was removed but the supraspinous ligament was preserved. The contact of two wings of Coflex with spinous process was set as binding contact, and the dentate part was ignored.

### Loading conditions

The fixed boundary condition restrained the inferior surface of the S1 segment in these models. A compressive load of 400 N and 10 Nm of momentum, rather than displacement on the most upper part of the spine models to simulate physiological activity, were applied on the superior surface of the L1 to generate compression, flexion, extension, rotation and lateral bending. In this study, range of motion (ROM) of each segment, intradiscal pressures and facet joints contact force of L4/L5 segment were examined in those 4 motions generated. Stress collection was mainly done by collecting the stress values of every node on the disc and nucleus pulposus of each segment in various positions and then calculating the average values. The variation of ROM was evaluated by the angular displacement. That is to say, the angle variation of superior surface line was confirmed according to the coordinate changes of the two nodes on the endplate of midsagittal view in states with 400 N compressive load and compressive load of 400 N + 10 Nm of momentum respectively. Each ROM was calculated three times, and finally the average value was collected. The formula is as follows:

$$ \mathrm{ROM}=\mid \frac{180}{\uppi}\times \arctan \frac{\mathrm{y}2-\mathrm{y}1}{\mathrm{x}2-\mathrm{x}1}-\frac{180}{\uppi}\times \arctan \frac{\mathrm{y}2\cdot -{\mathrm{y}1}^{{}^{\circ}}}{\mathrm{x}2\cdot -\mathrm{x}1\cdot}\mid $$

## Results

These models were validated before analysis of the result (Table 2). The stiffness result measured from the healthy model was compared with earlier biomechanical results from cadavers [14–17] and showed similar results. The difference between this study and Yamamoto’s study was not significantly. The difference is considered to occur due to the difference from the models details and selected subjects.

**Table 2:** Stiffness comparison with the results of the list literature

	Moment (Nm)	Anteflexion (N·m/°)	Postextension (N·m /°)	Left rotation (N·m /°)	Left bending (N·m /°)
Heth et al. [14]	10	1.1	2.35	1.33	2.61
Li et al. [15]	6	1.62	3.03	2.5	4.45
Liu et al. [16]	10	2.35	3.58	2.86	8.98
Yamamoto et al. [17]	10	1.75	3.22	2.44	5.66
This study	10	1.69	2.7	1.58	4.02
*P* value	/	0.957	0.274	0.296	0.372

The *p* values were determined with the one-simples T test

**Fig. 3:**
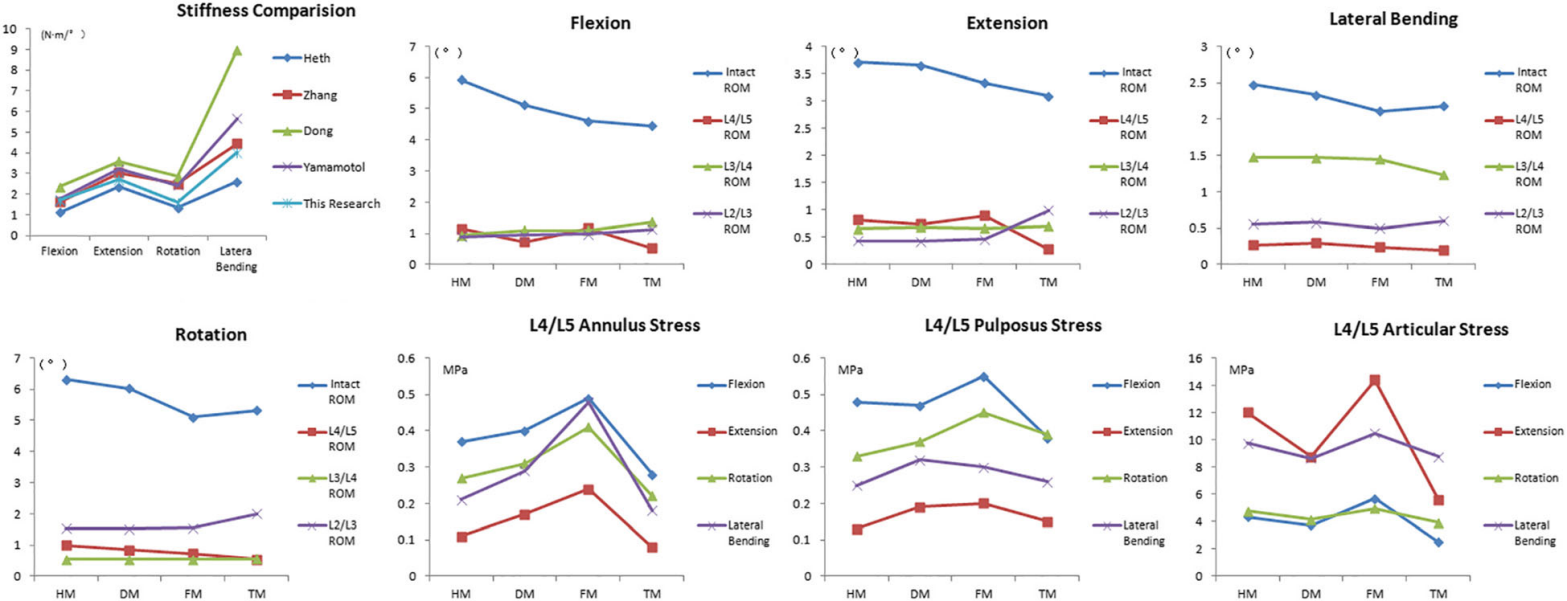
Stiffness conmparision results and ROM and von Mises stress distribution changes among various surgical models under flexion, extension, lateral bending, and axial rotation

Compared with the healthy model, the ROM of the total lumbar spine of the rest three models all decreased in the postures of anterior flexion, posterior extension, left bending, and left rotation. [see Additional file 1] The ROM of L4-L5 segment of Topping-off model decreased significantly by 28.39%、62.43%、30.82 and 36.45% in flexion, extension, axial rotation and lateral bending, while that of the fusion model increased by 38.31 and 21.70% in flexion and extension, when compared with degenerated model. L3-L4 segment and L2/L3 segment in Topping-off model respectively resulted in increase by 24.77% in flexion and 20.21, 130.23, and 32.45% in flexion, extension and axial rotation, while fusion model did not affect ROM of other segments compared with that of the degenerated model. Compared with degenerated model, the stress of annulus fibrosus, nucleus pulposus and articular process of fusion model all increased obviously in each active position, specially for flexion and extension, and the stress of the three elements in Topping-off model decreased significantly in anteflexion and extension position (Fig. 3).

## Discussion

The non-fusion surgery can minimize the influence on adjacent segments by preserving the motion of the lesion segments to prevent the occurrence of ASDeg. However, when faced with severe clinical situation of lumbar instability, osteoporosis and severe spinal stenosis, fusion is usually needed [5, 6, 13, 18]. The increase of movement and stress of adjacent segments after fusion is the main cause of ASDeg, moreover, for the degenerated adjacent disc, fusion may accelerate degeneration process, even result in symptomatic degeneration [19, 20], especially for those with indications of fusion and moderate degeneration in the superior adjacent disc(Pfirrmann grade II-IV) [21], the fusion segments should be minimized while achieving good clinical results. As a hybrid internal fixation technique, Topping-off technique may be a fair way to solve the situation [10, 22, 23].

Limited to the fact that the internal mechanical environment of the human body cannot be measured directly, the three-dimensional finite element analysis method is used to simulate the internal mechanical environment of the human body through the establishment of effective lumbar spine models. The biomechanical analysis of the entire lumbar after Topping-off were performed in the lumbosacral junction region where the biomechanical environment of lumbosacral region changed into a rigid lever consisted of pelvis, sacrum and L1-L5 segments together after L5-S1 fusion and then the stress and mobility of upper segments increased due to the relative stability of the pelvis and sacrum. There are a few studies on the changes of mechanical environment after Topping-off technique at present, nevertheless, the changes of mechanical environment of lumbosacral junction region with relatively concentrated stress and the influence of topping-off on the whole lumbar mechanical environment have rarely been referred. In addition, This study showed that, compared with the healthy model, the stress of annulus fibrosus and nucleus pulposus of L4-L5 in degenerated model increased in flexion, extension, axial rotation and bending position, while the ROM of each segment and the stress of posterior joints decreased. So, early disc degeneration may result in a change in the biomechanical state of the corresponding segments. Therefore, in order to study the effects of lumbar fusion and Topping-off on the superior segments, it was rational that the fusion model and Topping-off model were created based on the degenerated model, and then compared with the degenerated model. Previous studies have shown that the degeneration of discs mainly lies in the decrease of proteoglycan concentration and collagen fibrosis, resulting in an increase in the hardness of discs [24, 25]. So, the establishment of the moderate degenerated model was mainly achieved by increasing the elastic modulus of the annulus fibrosus, reducing the volume of the elastic matrix of the annulus fibrosus and reducing the elastic modulus of the nucleus pulposus.

Those results showed an significant increase in ROM of L4-L5 in the fusion model under different positions, especially in flexion, but no significant changes were observed in other segments. Therefore, the compensatory effect of lumbar motion after fusion mainly focused on the L4-L5 segment. Excessive activity results in the change of rotation center in the corresponding segment, which may not only tend to impair the annular fiber and endplate and lead to poor blood supplying, lower nutrition diffusivity and hydraulic permeability, but also influence the resulting forces in the facet joints, making for the resultant apoptosis and accelerated degeneration [26–29]. Several studies have shown that mechanical stimulation plays an important role in the regulation of disc biology and this has indicated that mechanical overloading is a risk factor for disc degeneration [30, 31]. As revealed in the results, Topping-off surgery significantly reduced the mobility of L4-L5 in the flexion and, to some extent, increased the ROM of L2-L4 segments, especially in flexion and extension position. Considering of the slight decrease in ROM of intact lumbar, it indicated that Coflex could not only limit the hyperactivity of the adjacent segments, but also distribute the compensatory effect of lumbar spine motion to the upper segments after fusion. And the intradiscal pressure was largest in the anteflexion position, which explained that thoracic disc frequently occurs in the anteflexion position in the clinic [32], and indirectly proved the validity of models.

In the lumbosacral junction region where the stress is relatively concentrated, increased disc and facet joints stress of the superior adjacent segment after L5-S1 fusion may lead to changes of biomechanical environment and structural disorders of disc, and make the intervertebral space narrow gradually, especially for the disc that has already degenerated [33]. Facet joints and disc are involved in maintaining stability and in the coupling movement of the spine in different directions. Hyperactivity may result in chronic pressure overload of disc and facet joints. Compared with degenerated model, pressure overload may result in pressure concentration, and then joints wear and remolding [34, 35]. Eventually, under the sustained influence of hyperactivity and pressure overload, moderate degenerated discs gradually develop into the degeneration of the whole segment. In this study, decreased ROM and stress of upper adjacent level indicated that Topping-off could protect facet joints and degenerated disc from hyperactivity and excessive stress,the hyperactivity of adjacent segments, but also reduce the stress of discs and facet joints and delay the progress of degenerated disc by compensating the lost motion of lumbar spine through other adjacent segments over time. In addition, in order to prevent the occurrence of ASDeg, clinicians should improve the surgical skills as much as possible, cause less damage to the superior articular capsule [36], and restore the lumbar kyphosis as far as possible [37].

## Conclusion

The results of the present models predict the effect of Topping-off surgery on the reduction of disc and facet joints stress and hyperactivity of the upper adjacent segment, and the ability of distributing the compensatory effect of lumbar spine motion to the upper segments after fusion. Thus it may protected the upper adjacent degenerated disc from progress to symptomatic degeneration. This study has some deficiencies which should combine with cadaveric experiments and incorporate simulation of paravertebral muscles, the role of which in maintaining stability of the spine can not be neglect, in the future studies.

## Supplementary information

**Additional file 1.** Stress and displacement of four models under different physiological loads.

## Data Availability

Some availability of data and materials were uploaded,and and corresponding author J Xu can be contacted to request the raw data.

## Abbreviations

ASDeg: Adjacent segment degeneration
DM: Degenerated Model
FM: Fusion Model
HM: Health Model
ROM: Range of motion
TM: Topping-off; Model
