# Supplementary material for: Biomechanical changes of degenerated adjacent segment and intact lumbar spine after lumbosacral topping-off surgery: a three-dimensional finite element analysis
Source: BMC Musculoskelet Disord. 2020 Feb 15;21:104. doi: 10.1186/s12891-020-3128-5 (PMC7023809; doi:10.1186/s12891-020-3128-5)
Supplement: Supplementary file 1 — Additional file 1. Stress and displacement of four models under different physiological loads. [file 12891_2020_3128_MOESM1_ESM.docx]

Stress and displacement of four models under different physiological loads

|  |  | The intact lumbar spine | L4/L5 | L3/L4 | L2/L3 | L4/L5annulus fibrosus | L4/L5nucleus pulposus | L4/L5articular process |
| --- | --- | --- | --- | --- | --- | --- | --- | --- |
|  |  | ROM | ROM | ROM | ROM | stress（MPa） | stress（MPa） | stress（MPa） |
| Flexion | Healthy model | 5.93 | 1.14 | 0.95 | 0.9 | 0.37 | 0.48 | 4.33 |
|  | Degenerative model | 5.13 | 0.72 | 1.09 | 0.94 | 0.4 | 0.47 | 3.75 |
|  | Fusion model | 4.61 | 1.17 | 1.1 | 0.98 | 0.49 | 0.55 | 5.68 |
|  | Topping-off model | 4.45 | 0.52 | 1.36 | 1.13 | 0.28 | 0.38 | 2.51 |
| Extension | Healthy model | 3.7 | 0.82 | 0.66 | 0.44 | 0.11 | 0.13 | 12 |
|  | Degenerative model | 3.65 | 0.74 | 0.68 | 0.43 | 0.17 | 0.19 | 8.76 |
|  | Fusion model | 3.33 | 0.9 | 0.67 | 0.46 | 0.24 | 0.2 | 14.4 |
|  | Topping-off model | 3.09 | 0.28 | 0.7 | 0.99 | 0.08 | 0.15 | 5.56 |
| Rotation | Healthy model | 6.32 | 1 | 0.55 | 1.54 | 0.27 | 0.33 | 4.77 |
|  | Degenerative model | 6.03 | 0.84 | 0.54 | 1.51 | 0.31 | 0.37 | 4.15 |
|  | Fusion model | 5.1 | 0.72 | 0.54 | 1.55 | 0.41 | 0.45 | 4.96 |
|  | Topping-off model | 5.33 | 0.54 | 0.56 | 2 | 0.22 | 0.39 | 3.92 |
| Bending | Healthy model | 2.48 | 0.27 | 1.48 | 0.56 | 0.21 | 0.25 | 9.77 |
|  | Degenerative model | 2.33 | 0.3 | 1.47 | 0.58 | 0.29 | 0.32 | 8.63 |
|  | Fusion model | 2.11 | 0.24 | 1.45 | 0.5 | 0.48 | 0.4 | 10.5 |
|  | Topping-off model | 2.18 | 0.2 | 1.23 | 0.6 | 0.18 | 0.26 | 8.77 |
